# Real-world data on the impact of dose reduction measures on radiation exposure in CT perfusion of the brain

**DOI:** 10.1016/j.ejro.2026.100759

**Published:** 2026-05-08

**Authors:** Friederike Lang, Thomas Stein, Till Schürmann, Martin Fiebich, Fabian Bamberg, Horst Urbach, Elias Kellner, Alexander Rau

**Affiliations:** a Department of Diagnostic and Interventional Radiology, Medical Center – University of Freiburg, Faculty of Medicine, University of Freiburg, Freiburg, Germany; b University of Applied Sciences Mittelhessen, Faculty of Life Science Engineering, Institute of Medical Physics and Radiation Protection, Gießen, Germany; c Department of Neuroradiology, Medical Center – University of Freiburg, Faculty of Medicine, University of Freiburg, Freiburg, Germany; d Medical Physics, Department of Diagnostic and Interventional Radiology, Medical Center – University of Freiburg, Faculty of Medicine, University of Freiburg, Freiburg, Germany

**Keywords:** Computed Tomography, Perfusion Imaging, Stroke Imaging, Cancer Risk, Radiation Dose

## Abstract

**Background:**

CT perfusion (CTP) imaging is a valuable tool for assessing cerebral blood flow. However, it is associated with high radiation doses, often exceeding those of non-contrast cranial CT. We evaluated the impact of different dose reduction approaches in CTP.

**Methods:**

We retrospectively included patients who underwent CTP in a stroke referral center. Imaging was performed using four different scanners. The protocols were iteratively optimized by decreasing the tube current time product through lowering the tube current while maintaining a constant tube rotation time. Additionally, the temporal sampling interval was increased from 1.5 to 3.0 s. Volume CT dose index (CTDI_vol_) was compared across the optimization steps. Effective doses were calculated and the theoretical lifetime cancer risk was estimated.

**Results:**

Radiation doses from 3812 cases with CTP (47.5% female, mean age 73 ± 14 years) were evaluated. Reducing the tube current by one third resulted in a dose decrease of 33.1%. Doubling the temporal sampling interval significantly reduced the dose by approximately half (47.3%-51.7%) across all scanners (all *p* < 0.001). Combining both adjustments on the same scanner led to an overall dose reduction of 67.7%. The lowest mean CTDI_vol_ and effective dose across all scanners were 69.2 ± 0.2 mGy and 0.61 ± 0.08 mSv, respectively. The estimated additional lifetime cancer risk was reduced by up to 67.7%, with greater absolute benefits in younger patients.

**Conclusion:**

Dose reduction measures allow for scanning CTP with exposure levels close to those of standard non-contrast CT.

## Highlights

• Large real-world cohort evaluating dose optimization measures in brain CT perfusion.
• Reduced tube current and longer sampling intervals lowered dose by up to 67.7%.
• Optimized protocols achieved radiation levels comparable to non-contrast head CT.
• Dose reduction translated into substantial decreases in estimated lifetime cancer risk.
• Benefits were most pronounced in younger patients undergoing CT perfusion imaging.

## 1 Introduction

Multimodal stroke computed tomography (CT) comprises non-contrast CT for the assessment of the brain parenchyma and CT angiography (CTA) to detect vessel occlusions [1]. CT perfusion (CTP) can be supplemented to assess the degree and extent of hypoperfused tissue and has been established for both diagnostics and clinical decision-making [2]. By repetitive scanning of the brain during a contrast agent bolus passage, CTP aims at separating salvageable tissue (i.e., the penumbra) and irreversibly damaged areas referred to as the infarct core [1], [3], [4].

Appropriate evaluation of CTP requires a sufficient contrast-to-noise ratio as well as a scan duration long enough to capture the complete bolus passage with sufficient temporal resolution to model the bolus anatomy [5]. This usually results in rather high radiation exposure and has led to a widespread consensus that achieving sufficient image quality in CTP is associated with high radiation dose [6]. Nevertheless, to date, no diagnostic reference level has been established for CTP, neither nationally nor internationally [7], [8], [9], [10], [11]. Although it is sometimes argued that radiation dose is of lesser importance in acute conditions such as stroke, the final diagnosis is not known a priori [12], [13]. Therefore, radiation exposure should be optimized for every examination in accordance with the ALARA principle (As Low As Reasonably Achievable).

In general, dose reduction strategies in CT encompass reducing the tube peak voltage (kVp) and the tube current time product (mAs). Early CTP protocols typically used a tube voltage of 120 kVp [14], but more recent studies have demonstrated that a reduction to 80 kVp or 70 kVp is feasible without compromising diagnostic accuracy [15], [16] while even enhancing imaging contrast [14]. In particular, using 70 kVp instead of 80 kVp can reduce the radiation dose by around 35% [15]. Due to the linear relationship between mAs and radiation dose, reducing the mAs leads to a proportional decrease in radiation exposure, albeit at the cost of increased image noise [17]. Riederer et al. [18] demonstrated that 80 mAs in combination with 80 kVp is still sufficient to maintain diagnostic accuracy for detecting perfusion deficits in large vessel occlusion. Similarly, Murphy et al. [19] showed that even a further reduction to 50 mAs is in principle feasible.

Current guidelines recommend a dynamic acquisition time of 40–90 s to fully capture the contrast bolus passage, with a minimum temporal sampling rate of 2–3 s [6], [20]. Many studies employed even shorter sampling intervals down to 1 s [21], [22], [23], [24]. Since reducing the number of acquisitions directly decreases radiation dose, an alternative approach to dose optimization is adjusting the sampling scheme. While some smaller studies have raised concerns that sampling intervals longer than 2 s may reduce the accuracy of perfusion measurements and potentially underestimate ischemic volume [25], [26], other studies have demonstrated that intervals of 3 s or even 6 s can be used without significantly compromising diagnostic accuracy. This adjustment can result in potential radiation dose reductions by a factor of up to 4 [21], [27].

Furthermore, non-uniform sampling schemes have been proposed as a dose reduction strategy, using wider sampling intervals at the beginning and end of the acquisition, while maintaining higher temporal resolution around the expected peak of contrast bolus arrival [16], [28], [29]. However, due to significant age-dependent and pathological variation in bolus arrival times—particularly in patients with delayed perfusion—such non-uniform schemes may fail to reliably capture the full bolus curve, especially in ischemic tissue [30]. For similar reasons, reducing the total scan duration below 45 s is not advisable, as it may result in bolus truncation, compromising the accuracy of perfusion analysis [30].

In this work, we present a retrospective analysis of real-world data on an iterative process of implementing dose reduction approaches through reduced mAs and increased temporal sampling interval in a large cohort of patients examined with different CT scanners.

## 2 Methods

The study was approved by the Institutional Review Board (Ethics Committee – University of Freiburg; EK 20-1047_1) and was carried out in accordance with the Declaration of Helsinki and its later amendments. Due to the retrospective nature of this study, the need for written informed consent was waived.

We retrospectively included patients who underwent CT perfusion imaging for suspected stroke between July 2021 and December 2024. Perfusion imaging was performed with four different CT scanners (all Siemens Healthineers, Forchheim, Germany): Somatom Definition AS (Scanner A), Somatom Definition Flash (Scanner B), Somatom Force (Scanner C), or Somatom goTop (Scanner D), with scanner availability varying throughout the period of analysis. All CTP series were acquired in the axial scan mode without gantry tilt. The dose optimization strategies were implemented as part of an iterative process. While the original protocol for each scanner was designated as ‘initial’, ’optimization 1’ referred to a reduction in tube current and ‘optimization 2’ to an increase in the temporal sampling interval, while keeping the total scan time nearly constant. Finally, ‘optimization 3’ involved an adjustment of the number of scans, effectively changing the overall scan time. Depending on the initial scan protocol, not every optimization step was applied to every scanner. All perfusion scans were processed using the VEOcore software (VEObrain GmbH, Freiburg, Germany, https://www.veobrain.com).

### 2.1 Scanning protocols and iterative dose reduction approach

Scanner A, launched in 2007 [31], was used during the entire period. Parameters for the initial protocol were as follows: 80 kVp, 16 × 1.20 mm collimation, 8.5 cm scan length, tube rotation time of 0.3 s, and spiral mode. Applying optimization 1, the mAs was reduced from 180 mAs to 120 mAs by the adjustment of the tube current while maintaining constant rotation time. The second optimization step involved increasing the temporal sampling interval from 1.5 s to 3.0 s, which simultaneously resulted in the reduction of the number of scans from 30 to 16, leading to scan durations of 45.0 s and 48.0 s, respectively.

Scanner B, introduced in 2009 [31], was used from July 2021 to February 2024 with 80 kVp, 90 mAs, 32 × 1.20 mm collimation, 9.5 cm scan length, a tube rotation time of 0.285 s, and spiral mode. For optimization, the temporal sampling interval was increased from 1.5 s to 3.0 s, which led to a decrease in the number of acquisitions from 27 to 15. This resulted in scan durations of 40.5 s and 45.0 s, respectively.

Scanner C, released in 2013 [31], was in use from October 2021 to December 2024. CTP data was acquired using 70 kVp, 100 mAs, 48 × 1.20 mm collimation, 10.8 cm scan length, a tube rotation time of 0.25 s, and spiral mode. The initial protocol acquired 28 dynamic scans with a temporal sampling interval of 1.5 s. Optimization involved increasing the temporal sampling interval to 3.0 s, initially with 16 scans, and later with 15 scans. This resulted in total scan durations of 48.0 s and 45.0 s for the optimized protocols, compared to 42.0 s in the original protocol. On Scanner C, an adapted protocol was used for patients with low cardiac output, consisting of 22 scans with a temporal sampling interval of 3.0 s, resulting in a total scan duration of 66.0 s.

Scanner D, which was launched in 2018, was a temporary replacement, available for only one month in 2024. Here, only a single protocol with the optimized temporal sampling interval was applied. Images were acquired at 70 kVp, 64 × 0.60 mm collimation, scan length of 3.3 cm, tube rotation time of 0.5 s, and sequential mode. Unlike the other scanners, tube current varied between the individual patients. In total, 16 images were acquired using a temporal sampling interval of 3.0 s over a scan duration of 48.0 s.

### 2.2 Radiation dose assessment

Radiation exposure was assessed using the volume CT dose index (CTDI_vol_) and dose length product (DLP) from each acquired CTP, which were obtained from the dose management system (DoseM INFINITT Europe GmbH, Frankfurt am Main, Germany). The effective dose, an estimate of the overall biological risk, was derived from the DLP using the age- and sex-dependent conversion factor *k* from Deak et al. [32], based on ICRP 103 recommendations. Since no specific factor exists for 70 kVp, the value for 80 kVp (k = 0.0018 mSv/mGy·cm) was used instead.

For all examinations, the achieved dose reduction was converted into a reduction of the estimated additional total lifetime cancer risk using the values provided by Wall et al. [33]. Since total lifetime cancer risk depends on age at exposure and sex, the mean effective dose was calculated separately for each age group according to the grouping scheme by Wall et al. [33]. Because no value was provided for the age group of 100–109 years, the value for the 90–99-year age group was applied instead. If no patients in the respective age group were examined, no lifetime cancer risk was calculated.

### 2.3 Statistical analysis

Data analysis was performed using Python (version 3.11.5). Normal distribution was assessed through visual inspection using histograms. Due to the low variability and an approximately symmetric distribution, mean ± standard deviation was reported for age, CTDI_vol_, and effective dose. For significance testing, two-sample *t*-test was applied. A two-sided *p*-value of less than 0.05 was considered statistically significant.

## 3 Results

### 3.1 Patient characteristics

In total, 3812 cases (47.5% female, mean age 73 ± 14 years, [range: 13–102 years]) were retrospectively identified, of which 556 were examined with scanner A, 2310 with scanner B, 906 with scanner C, and 40 with scanner D. Detailed patient characteristics across iterative optimization steps are shown in Table 1.

**Table 1:** Patient demographics by CT scanner and optimization step.

**Scanner**	**Patients (n)**	**Age (years) ± sd [min-max]**	**Sex (female) (%)**
**Scanner A** Initial Optimization 1 Optimization 2	556 56 113 387	69 ± 14 [13–97] 74 ± 13 [24–95] 67 ± 16 [13–97] 68 ± 14 [20–97]	51.8 33.9 48.7 55.3
**Scanner B** Initial Optimization 2	2310 693 1617	74 ± 14 [15−100] 74 ± 14 [15−100] 74 ± 14 [18−100]	46.4 47.5 45.9
**Scanner C** Initial Optimization 2 Optimization 3 Low cardiac output	906 41 619 193 53	74 ± 14 [20−102] 76 ± 11 [52–92] 73 ± 15 [20−102] 75 ± 10 [43–96] 76 ± 11 [35–92]	47.0 56.1 47.8 47.7 28.3
**Scanner D**	40	78 ± 14 [35−100]	40.0

Detailed overview of the number of patients*,* mean age ± standard deviation (sd) and range as well as sex for each scanner and optimization step. ‘Initial’ refers to the initial scan protocol without optimization. ’Optimization 1’ refers to a reduction in tube current, ‘optimization 2’ to an increase in the temporal sampling interval, and ‘optimization 3’ to an adaptation of the number of scans.

### 3.2 Radiation dose assessment

The reduction of mAs for scanner A by one-third, from 180 mAs to 120 mAs, resulted in a corresponding reduction of the CTDI_vol_ from 261.6 mGy to 175.1 mGy and effective dose from 4.46 mSv to 2.98 mSv, respectively. Doubling the sampling interval while keeping the total scan duration nearly constant reduced the radiation dose by half, as this inherently led to a reduction in the number of scans by the same factor. For scanner A, this resulted in a reduction of mean CTDI_vol_ from 175.1 mGy to 84.6 mGy (52%), and the mean effective dose was reduced from 2.99 mSv to 1.44 mSv (52%). The combination of optimization 1 and optimization 2 resulted in an overall reduction of radiation dose to approximately one-third compared to the initial protocol.

For scanner B, optimization reduced the mean CTDI_vol_ from 209.8 mGy to 106.3 mGy and the effective dose from 4.45 mSv to 2.26 mSv, resulting in a radiation dose reduction of about 49% compared to the initial protocol.

For scanner C, optimization 2 and 3 were applied. Starting from a mean CTDI_vol_ of 137.2 mGy and a mean effective dose of 3.66 mSv, optimization steps reduced the mean CTDI_vol_ to 69.2 mGy and the mean effective dose to 1.93 mSv. The protocol for the patients with low cardiac output covers a longer scan duration with a mean CTDI_vol_ of 101.5 mGy and a corresponding mean effective dose of 2.83 mSv.

All applied optimization steps for all CT scanners led to significant reductions in radiation dose (all *p* < 0.001).

The implementation of the reduced temporal sampling for scanner D, which was not present in the manufacturer’s preset, resulted in a mean CTDI_vol_ of 88.2 mGy and a mean effective dose of 0.61 mSv.

**Fig. 1:**
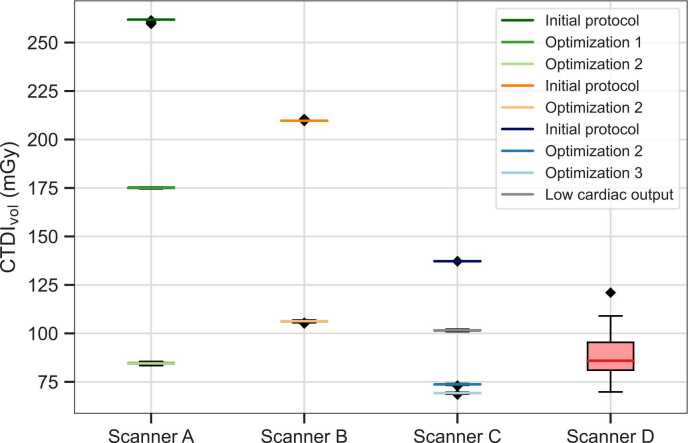
Impact of dose optimization strategies on CTDI_vol_ across all CT scanners used. Each boxplot represents an optimization step, with the boxplots showing the highest CTDI_vol_ for each scanner representing the initial protocol.

**Fig. 2:**
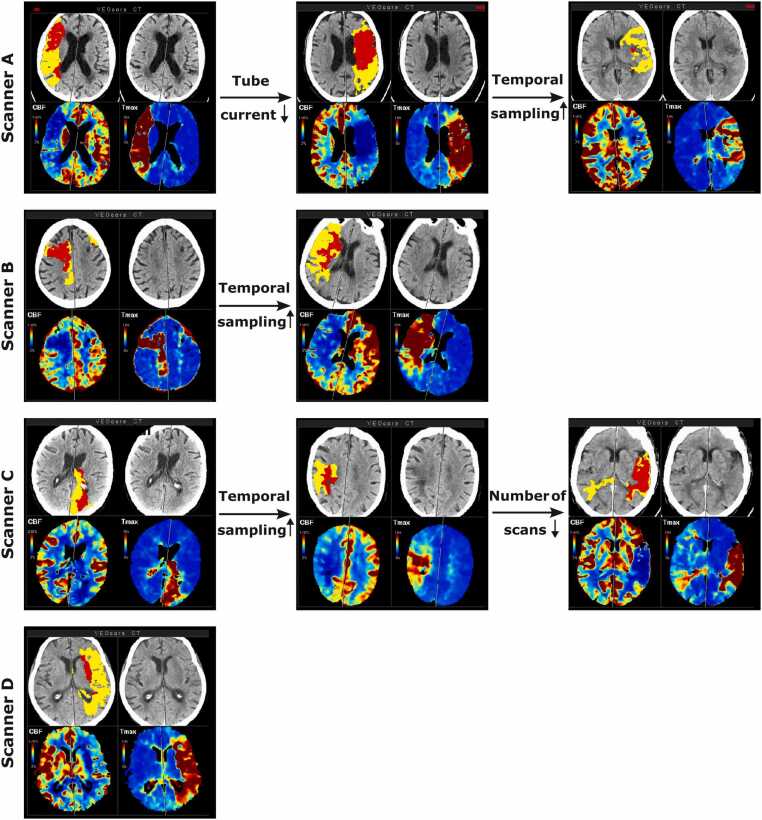
Exemplary automated processing outputs of the perfusion scans for each scanner and applied optimization step.

A detailed overview of the individual optimization steps and the corresponding radiation doses is shown in Table 2 and visualized in Fig. 1. Exemplary processed perfusion data across the optimization steps are shown in Fig. 2.

**Table 2:** Summary of imaging parameters modified for protocol optimization and impact on radiation dose, expressed as volume CT dose index (CTDI_vol_) and effective dose.

**Scanner**	**Tube current time product (mAs)**	**Temp. sampling interval (s)**	**Time points (n)**	**Scan duration (s)**	**CTDI**_**vol**_ **(mGy)**	**Effective Dose (mS)**
**Scanner A**						
Initial Optimization 1 Optimization 2	180 120 120	1.5 1.5 3.0	30 30 16	45.0 45.0 48.0	261.6 ± 0.6 175.1 ± 0.2 84.6 ± 0.4	4.46 ± 0.01 2.98 ± < 0.01 1.44 ± < 0.01
**Scanner B**						
Initial Optimization 2	90 90	1.5 3.0	27 15	40.5 45.0	209.8 ± 0.3 106.3 ± 0.3	4.45 ± < 0.01 2.26 ± < 0.01
**Scanner C**						
Initial Optimization 2 Optimization 3 Low cardiac output	100 100 100 100	1.5 3.0 3.0 3.0	28 16 15 22	42.0 48.0 45.0 66.0	137.2 ± < 0.01 73.7 ± 0.2 69.2 ± 0.2 101.5 ± 0.3	3.66 ± < 0.01 2.06 ± < 0.01 1.93 ± < 0.01 2.83 ± < 0.01
**Scanner D**	132 ± 16 [104–181]	3.0	16	48.0	88 ± 11	0.61 ± 0.08

‘Initial’ refers to the initial scan protocol without optimization. ’Optimization 1’ refers to a reduction in tube current, ‘optimization 2’ to an increase in the temporal sampling interval, and ‘optimization 3’ to an adaptation of the number of scans.

### 3.3 Theoretical cancer risk reduction

Along with the observed reduction in radiation dose, protocol optimization was associated with a theoretical reduction in additional lifetime cancer risk for both males and females across all age groups. For males in the age group of 70–79 years, which represented the mean age of the reported dataset, the additional lifetime cancer risk could be reduced by 67.7% (from 0.0062% to 0.0020%) using scanner A, by 48.4% (from 0.0062% to 0.0032%) using scanner B, and by 47.1% (from 0.0051% to 0.0027%) using scanner C. For scanner D, only a single protocol was used, which resulted in an estimated additional lifetime cancer risk of 0.0010% for the same age group. For females, the additional lifetime cancer risk was generally lower than that of male patients. However, for female patients in the age group of 70–79 years, protocol optimization resulted in a reduction of the additional lifetime cancer risk of 68.9% (from 0.0045% to 0.0014%) using scanner A, 48.9% (from 0.0045% to 0.0023%) using scanner B, and 48.7% (from 0.0037% to 0.0019%) using scanner C. For scanner D, it was determined to be 0.0006%. The underlying data used to calculate the additional lifetime cancer risk can be found in Tables S1 and S2 of the Supplementary Material.

**Fig. 3:**
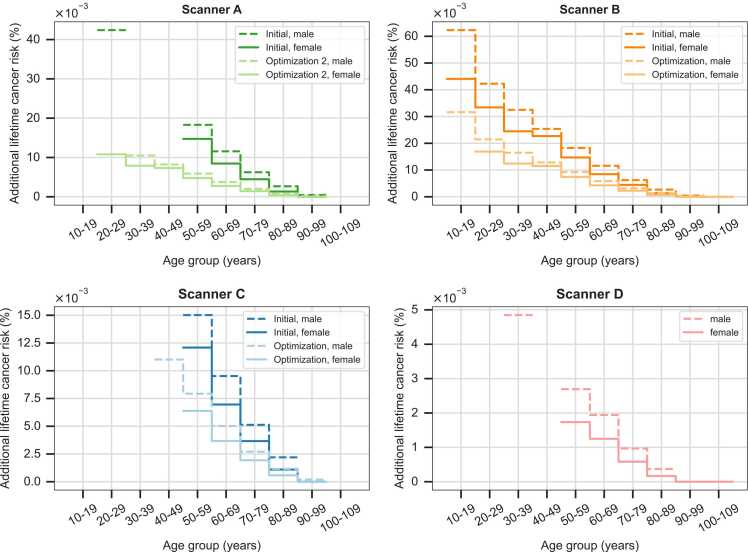
Reduction in additional lifetime cancer risk due to protocol optimization across the four CT scanners as a function of age. Since the conversion factors were provided only for age groups, the additional lifetime cancer risk is represented as a stepwise function.

Given that the additional lifetime cancer risk is higher in younger age groups, for males aged between 10–19 years, protocol optimization on CT scanner B led to a decrease in the additional lifetime cancer risk of about 49.3% (from 0.0623% to 0.0316%), which is about 10 times higher than for the mean age group. A detailed presentation of the additional lifetime cancer risk depending on age and CT scanner is shown in Fig. 3.

## 4 Discussion

CTP is often associated with high radiation exposure, a concern highlighted by the US Food and Drug Administration (FDA) in 2009 [34]. Various approaches have been suggested and implemented in clinical routine, such as the reduction in mAs or the increase in the temporal sampling interval, while maintaining a constant total scan duration. In this retrospective study we evaluated the iterative implementation of radiation dose reduction measures in CTP based on a large real-world dataset acquired across multiple CT scanners in routine clinical practice, and demonstrated a measurable improvement in patient safety through effective dose optimization.

Both CTDI_vol_ and effective dose could be significantly reduced by 67.7% for scanner A, 49.4% for scanner B, and 47.3% when using scanner C. The optimized values of 1.44 mSv, 2.26 mSv, and 1.93 mSv for scanners A, B, and C, respectively, are comparable with the effective dose for standard non-contrast head CT, which is reported to range between 1.22 mSv and 2 mSv [35], [36], [37]. Although scanner A operates at a higher mAs and a higher number of scans compared to scanner B, the use of a thicker thin filter and a smaller collimation resulted in lower CTDI_vol_ and effective dose for scanner A. A significant reduction in comparison to the initial protocol was also obtained for the protocol with a longer scan duration of 66 s, which is used for patients with low cardiac output. Both underscore that our dose optimization approaches can be applied independently of other acquisition parameters, with the latter affecting only the absolute dose level. The comparatively low effective dose of 0.61 ± 0.08 mSv for CT scanner D can be attributed to the use of the sequential scan mode and the substantially shorter *z*-axis coverage, which limits the comparability with the other three scanners. Specifically, the shorter *z*-coverage might hamper diagnostic value as substantial parts of the brain are excluded from the field of view. In addition to that, sequential scan mode is still used on some older systems but is becoming rare, so results from scanner D may have limited and decreasing clinical relevance.

According to the CTP protocols provided by the American Association of Physicists in Medicine (AAPM) for various manufacturers, the CTDI_vol_ for CTP ranges from 66 mGy to 452 mGy, depending on the CT scanner and acquisition mode used, with the vast majority (60%) exceeding 200 mGy [20]. In contrast, Christensen and Lansberg [38] reported that a minimum CTDI_vol_ of 300 mGy is required to achieve both adequate image quality and an accurate volume estimate. However, our real-world optimization in clinical routine demonstrates that a dose reduction far below 300 mGy is possible. Forbrig et al. [39] reported an optimized CTDI_vol_ of 141.1 mGy using the same dual-source CT scanner as our scanner C. This is comparable to the radiation dose of our initial protocol and approximately twice as high compared to the final optimization step in our study. Nicolas et al. [40] reported a CTDI_vol_ of 104 ± 2 mGy, using the same scanner with 170 mAs, 70 kVp, and again 1.5 s temporal sampling interval. Based on these studies, our results highlight the potential for dose reduction by adjusting the mAs and especially the temporal sampling rate. While we did not explicitly assess diagnostic accuracy in this study, its preservation has been well demonstrated in prior detailed simulation studies that directly compared different sampling intervals. These studies showed that increasing the interval to 3.0 s does not significantly affect CTP metrics, image quality, or treatment decisions [21], [27]. Moreover, all optimization steps were conducted iteratively and in close collaboration between neuroradiologists, neurologists, medical physicists, and vendors of CTP processing software to ensure that diagnostic image quality was maintained throughout the process. This cooperative collaboration also explains why no further reduction of the kVp value was sought. It was already set to a comparable low value in the initial protocols, and a further reduction would have led to increased image noise.

A study by Smith-Bindman et al. [41] estimated that CT imaging accounts for approximately 5% of all newly diagnosed cancers each year, highlighting the importance of optimizing radiation dose reduction measures. In this study, dose reduction strategies for CT perfusion of the brain translate into a significant decrease in the estimated total lifetime cancer risk. For CT scanner A, the additional total lifetime cancer risk in all age groups could be reduced by a factor of 3.1, for CT scanners B and C by factors of 2.0 and 1.9, respectively for males and females. This implies that implementing dose reduction strategies for patients in the 70–79 age group could prevent up to 4 cases of cancer per 100 000 examinations in males and 3 cases per 100 000 examinations in females. The potential benefits of dose reduction are further increased for the younger age groups. For male patients in the age group of 10–19 years, protocol optimization could potentially help to avoid more than 30 cases per 100 000 examinations when using scanner B.

Beyond the increased lifetime cancer risk, exposure of the eye lens to ionizing radiation is a critical factor, as the eye lens is one of the most radiosensitive tissues and even low doses can increase the risk of radiation-induced cataracts [42], [43], [44]. Despite efforts to exclude the lens from the CTP scan range, exposure of the lens cannot be entirely ruled out [45], [46], which underscores the importance of dose reduction strategies to mitigate this risk.

Importantly, the same logic can be applied in reverse: with lower-dose protocols now available, it is safer to include younger patients in routine emergency stroke imaging workflows that incorporate CTP, allowing them to benefit from its added diagnostic value without compromising safety.

Finally, the dose optimization in our study represents just one step in an ongoing, iterative process toward improving patient safety. Simulation data suggest that even a temporal sampling interval of 6.0 s is feasible [27], and with continued advancements in reconstruction algorithms, image processing, and artificial intelligence, further dose reductions could become viable. In addition, future work may address the challenge of contrast agent dose reduction, further enhancing the safety and accessibility of CTP in clinical practice.

### 4.1 Limitations

While we report a large-scale real-world dataset, our study is limited by its retrospective design. No independent reference standard was available, and the optimization process reflects an iterative, non-standardized implementation of dose reduction rather than a uniform protocol optimization. The parameter optimizations were intentionally defensive, and all examinations were performed on scanners from a single manufacturer, which may limit generalizability. Nevertheless, the applied dose-reduction strategies are transferable to other systems, and further methodological improvements and prospective, multi-center studies will be essential to advance optimization efforts.

## 5 Conclusion

Our real-world large-scale data on iterative dose reduction in CTP highlights the substantial potential of protocol optimization to significantly lower patient radiation exposure. This challenges the long-standing assumption that high radiation exposure is necessary for sufficient image quality in CTP, as we achieved effective doses comparable to those of a standard non-contrast head CT. The potential reduction in lifetime cancer risk is particularly relevant for younger patients.

## CRediT authorship contribution statement

**Alexander Rau:** Conceptualization, Data curation, Formal analysis, Investigation, Resources, Supervision, Validation, Writing – original draft, Writing – review & editing. **Elias Kellner:** Conceptualization, Formal analysis, Investigation, Methodology, Software, Writing – original draft, Writing – review & editing. **Horst Urbach:** Data curation, Resources, Writing – review & editing. **Thomas Stein:** Data curation, Supervision, Writing – review & editing. **Till Schürmann:** Data curation, Formal analysis, Writing – review & editing. **Friederike Lang:** Conceptualization, Data curation, Formal analysis, Investigation, Methodology, Project administration, Resources, Software, Validation, Visualization, Writing – original draft, Writing – review & editing. **Fabian Bamberg:** Resources, Writing – review & editing. **Martin Fiebich:** Validation, Writing – review & editing.

## Ethics

Institutional Review Board approval was obtained (Ethics Committee – University of Freiburg; EK 20–1047_1). Written informed consent was not required for this study because of the retrospective nature of the study and therefore waived by the Institutional Review Board.

## Ethical approval

Institutional Review Board approval was obtained (Ethics Committee – University of Freiburg; EK 20–1047_1).

## Informed consent

Written informed consent was not required for this study because of the retrospective nature of the study and therefore waived by the Institutional Review Board.

## Guarantor

The scientific guarantor of this publication is Elias Kellner.

## Statistics and biometry

No complex statistical methods were necessary for this paper.

## Methodology

Methodology:

• retrospective
• cross sectional study
• performed at one institution

## Study subjects or cohorts overlap

Not applicable.

## Funding

The authors state that this work has not received any funding.

## Declaration of competing interest

EK is a shareholder of and receives payments from VEObrain GmbH. The other authors of this manuscript declare no relationships with any companies, whose products or services may be related to the subject matter of the article.

## Data Availability

Data is available from the authors upon reasonable request.

## References

[1] Powers W.J., Rabinstein A.A., Ackerson T., Adeoye O.M., Bambakidis N.C., Becker K., Biller J., Brown M., Demaerschalk B.M., Hoh B., others (2019). Guidelines for the early management of patients with acute ischemic stroke: 2019 update to the 2018 guidelines for the early management of acute ischemic stroke: a guideline for healthcare professionals from the American Heart Association/American Stroke Association. Stroke.

[2] Campbell B.C., Weir L., Desmond P.M., Tu H.T., Hand P.J., Yan B., Donnan G.A., Parsons M.W., Davis S.M. (2013). CT perfusion improves diagnostic accuracy and confidence in acute ischaemic stroke. J. Neurol. Neurosurg. Psychiatry.

[3] Albers G.W., Marks M.P., Kemp S., Christensen S., Tsai J.P., Ortega-Gutierrez S., McTaggart R.A., Torbey M.T., Kim-Tenser M., Leslie-Mazwi T., others (2018). Thrombectomy for stroke at 6–16 h with selection by perfusion imaging. N. Engl. J. Med..

[4] Boulanger J., Lindsay M., Gubitz G., Smith E., Stotts G., Foley N., Bhogal S., Boyle K., Braun L., Goddard T., others (2018). Canadian stroke best practice recommendations for acute stroke management: prehospital, emergency department, and acute inpatient stroke care, update 2018. Int. J. Stroke.

[5] Demeestere J., Wouters A., Christensen S., Lemmens R., Lansberg M.G. (2020). Review of perfusion imaging in acute ischemic stroke: from time to tissue. Stroke.

[6] Bundesärztekammer, Leitlinie der Bundesärztekammer zur Qualitätssicherung in der Computertomographie, (2023). 〈https://www.bundesaerztekammer.de/fileadmin/user_upload/BAEK/Themen/Qualitaetssicherung/_Bek_BAEK_Leitlinie_Computertomographie_ONLINE_KORR_Vers_25_05_2023.pdf〉.

[7] Bundesamt für Strahlenschutz, Bekanntmachung der aktualisierten diagnostischen Referenzwerte für diagnostische und interventionelle Röntgenanwendungen, (2022). 〈https://www.bfs.de/SharedDocs/Downloads/BfS/DE/fachinfo/ion/drw-roentgen.pdf?__blob=publicationFile&v=1〉. 10.1055/a-1813-3116 35817033

[8] Kanal K.M., Butler P.F., Sengupta D., Bhargavan-Chatfield M., Coombs L.P., Morin R.L. (2017). US diagnostic reference levels and achievable doses for 10 adult CT examinations. Radiology.

[9] Bundesamt für Gesundheit BAG, Diagnostische Referenzwerte in der Computertomografie, (2018). 〈https://www.bag.admin.ch/de/diagnostische-referenzwerte-im-strahlenschutz〉.

[10] UK Health Security Agency, National Diagnostic Reference Levels (NDRLs) from 20 November 2024, (2024). 〈https://www.gov.uk/government/publications/diagnostic-radiology-national-diagnostic-reference-levels-ndrls/ndrl〉.

[11] Dedulle A., Van Slambrouck K., Fremout A. (2021). Niv. De. R. éF. érence Diagn. Natx. En. Radiol. Onzième itéRatio pour Les. Exam. CT.

[12] Arora K., Gaekwad A., Evans J., O’Brien W., Ang T., Garcia-Esperon C., Blair C., Edwards L.S., Chew B.L., Delcourt C., others (2022). Diagnostic utility of computed tomography perfusion in the telestroke setting. Stroke.

[13] Biesbroek J., Niesten J., Dankbaar J., Biessels G., Velthuis B., Reitsma J., Van Der Schaaf I. (2013). Diagnostic accuracy of CT perfusion imaging for detecting acute ischemic stroke: a systematic review and meta-analysis. Cerebrovasc. Dis..

[14] Wintermark M., Maeder P., Verdun F.R., Thiran J.-P., Valley J.-F., Schnyder P., Meuli R. (2000). Using 80 kVp versus 120 kVp in perfusion CT measurement of regional cerebral blood flow. Am. J. Neuroradiol..

[15] Li Z., Li H., Zhang K., Li W., Chen X., Wu B., Song B. (2014). Improvement of image quality and radiation dose of CT perfusion of the brain by means of low-tube voltage (70 KV). Eur. Radio..

[16] Fang X.K., Ni Q.Q., Schoepf U.J., Zhou C.S., Chen G.Z., Luo S., Fuller S.R., De Cecco C.N., Zhang L.J., Lu G.M. (2016). Image quality, radiation dose and diagnostic accuracy of 70 kVp whole brain volumetric CT perfusion imaging: a preliminary study. Eur. Radio..

[17] Othman A.E., Afat S., Brockmann M.A., Nikoubashman O., Brockmann C., Nikolaou K., Wiesmann M. (2016). Radiation dose reduction in perfusion CT imaging of the brain: A review of the literature. J. Neuroradiol..

[18] Riederer I., Zimmer C., Pfeiffer D., Wunderlich S., Poppert H., Rummeny E.J., Huber A. (2018). Radiation dose reduction in perfusion CT imaging of the brain using a 256-slice CT: 80 mAs versus 160 mAs. Clin. Imaging.

[19] Murphy A., So A., Lee T.-Y., Symons S., Jakubovic R., Zhang L., Aviv R.I. (2014). Low dose CT perfusion in acute ischemic stroke. Neuroradiology.

[20] AAPM, Adult Brain Perfusion CT Protocols, (2016). 〈https://www.aapm.org/pubs/CTProtocols/documents/AdultBrainPerfusionCT.pdf〉.

[21] Wiesmann M., Berg S., Bohner G., Klingebiel R., Schöpf V., Stoeckelhuber B., Yousry I., Linn J., Missler U. (2008). Dose reduction in dynamic perfusion CT of the brain: effects of the scan frequency on measurements of cerebral blood flow, cerebral blood volume, and mean transit time. Eur. Radio..

[22] Abels B., Klotz E., Tomandl B., Villablanca J., Kloska S., Lell M. (2011). CT perfusion in acute ischemic stroke: a comparison of 2-second and 1-second temporal resolution. Am. J. Neuroradiol..

[23] Ma G., Cao Y.-Z., Shen G.-C., Lu S.-S., Zhang Y.-X., Zhang Y., Shi H.-B., Xu X.-Q., Wu F.-Y. (2023). CT perfusion with increased temporal sampling interval to predict target mismatch status in patients with acute ischemic stroke. Neuroradiology.

[24] Ioannidis G.S., Christensen S., Nikiforaki K., Trivizakis E., Perisinakis K., Hatzidakis A., Karantanas A., Reyes M., Lansberg M., Marias K. (2021). Cerebral CT perfusion in acute stroke: the effect of lowering the tube load and sampling rate on the reproducibility of parametric maps. Diagnostics.

[25] Kloska S.P., Fischer T., Sauerland C., Buerke B., Dziewas R., Fischbach R., Heindel W. (2010). Increasing sampling interval in cerebral perfusion CT: limitation for the maximum slope model. Acad. Radio..

[26] Kämena A., Streitparth F., Grieser C., Lehmkuhl L., Jamil B., Wojtal K., Ricke J., Pech M. (2007). Dynamic perfusion CT: optimizing the temporal resolution for the calculation of perfusion CT parameters in stroke patients. Eur. J. Radio..

[27] Rau A., Reisert M., Stein T., Mueller-Peltzer K., Rau S., Bamberg F., Taschner C.A., Urbach H., Kellner E. (2024). Impact of temporal resolution on perfusion metrics, therapy decision, and radiation dose reduction in brain CT perfusion in patients with suspected stroke. Neuroradiology.

[28] Cros M., Geleijns J., Joemai R.M., Salvado M. (2016). Perfusion CT of the brain and liver and of lung tumors: use of Monte Carlo simulation for patient dose estimation for examinations with a cone-beam 320-MDCT scanner. Am. J. Roentgenol..

[29] Shankar J.J.S., Lum C. (2011). Whole brain CT perfusion on a 320-slice CT scanner. Indian J. Radiol. Imaging.

[30] Stein T., Kellner E., Mueller-Peltzer K., Elsheikh S., Reisert M., Hosp J.A., Bamberg F., Urbach H., Rau A. (2024). Assessing bolus peak position in CT perfusion: High variance persisting despite age-dependency in a large cohort. Eur. J. Radio..

[31] Siemens Healthcare GmbH, Siemens Healthineers Historical Institute. The history of computed tomography at Siemens Healthineers, (2021). 〈https://cdn0.scrvt.com/39b415fb07de4d9656c7b516d8e2d907/009f90a9278c67f0/61c32a945640/7610_CC_MedMuseum_History_CT_book_eng_FINAL-1-.pdf〉.

[32] Deak P.D., Smal Y., Kalender W.A. (2010). Multisection CT protocols: sex-and age-specific conversion factors used to determine effective dose from dose-length product. Radiology.

[33] Wall B.F., Haylock R., Jansen J.T.M., Hillier M.C., Hart D., Shrimpton P.C. (2011).

[34] Wintermark M., Lev M. (2010). FDA investigates the safety of brain perfusion CT. AJNR Am. J. Neuroradiol..

[35] Jaffe T.A., Hoang J.K., Yoshizumi T.T., Toncheva G., Lowry C., Ravin C. (2010). Radiation dose for routine clinical adult brain CT: variability on different scanners at one institution. Am. J. Roentgenol..

[36] McCollough C.H., Bushberg J.T., Fletcher J.G., Eckel L.J. (2015). in: Mayo Clin. Proc.

[37] Smith-Bindman R., Wang Y., Chu P., Chung R., Einstein A.J., Balcombe J., Cocker M., Das M., Delman B.N., Flynn M., others (2019). International variation in radiation dose for computed tomography examinations: prospective cohort study. Bmj 364.

[38] Christensen S., Lansberg M.G. (2019). CT perfusion in acute stroke: practical guidance for implementation in clinical practice. J. Cereb. Blood Flow. Metab..

[39] Forbrig R., Trumm C.G., Reidler P., Kunz W.G., Dimitriadis K., Kellert L., Rückel J., Liebig T., Stahl R. (2024). Optimizing Radiation Dose and Image Quality in Stroke CT Protocols: Proposed Diagnostic Reference Levels for Multiphase CT Angiography and Perfusion Imaging. Diagnostics.

[40] Nicolas P.M., Maksoud Z., Nacul N.G., Akkurt B.H., Mannil M., Musigmann M. (2024). Diagnostic value of routine CT perfusion imaging for radiology residents. Sci. Rep..

[41] Smith-Bindman R., Chu P.W., Firdaus H.A., Stewart C., Malekhedayat M., Alber S., Bolch W.E., Mahendra M., de González A.B., Miglioretti D.L. (2025). Projected lifetime cancer risks from current computed tomography imaging. JAMA Intern. Med..

[42] Ainsbury E., Bouffler S., Dörr W., Graw J., Muirhead C., Edwards A., Cooper J. (2009). Radiation cataractogenesis: a review of recent studies. Radiat. Res..

[43] Stewart F., Akleyev A., Hauer-Jensen M., Hendry J., Kleiman N., Macvittie T., Aleman B., Edgar A., Mabuchi K., Muirhead C., others (2012). ICRP publication 118: ICRP statement on tissue reactions and early and late effects of radiation in normal tissues and organs–threshold doses for tissue reactions in a radiation protection context. Ann. ICRP.

[44] Chodick G., Bekiroglu N., Hauptmann M., Alexander B.H., Freedman D.M., Doody M.M., Cheung L.C., Simon S.L., Weinstock R.M., Bouville A., others (2008). Risk of cataract after exposure to low doses of ionizing radiation: a 20-year prospective cohort study among US radiologic technologists. Am. J. Epidemiol..

[45] Lopez-Rendon X., Stratis A., Zhang G., Coudyzer W., Develter W., Bogaerts R., Bosmans H., Zanca F. (2020). Peak skin and eye lens radiation dose from brain perfusion CT: CTDIvol and Monte Carlo based estimations. Eur. J. Radio..

[46] Schilham A., van der Molen A.J., Prokop M., de Jong H.W. (2010). Overranging at multisection CT: an underestimated source of excess radiation exposure. Radiographics.

