# Supplementary material for: Real-world data on the impact of dose reduction measures on radiation exposure in CT perfusion of the brain
Source: Eur J Radiol Open. 2026 May 8;16:100759. doi: 10.1016/j.ejro.2026.100759 (PMC13186064; doi:10.1016/j.ejro.2026.100759)
Supplement: Supplementary file 1 — Supplementary material [file mmc1.docx]

# Supplemental Material

**Table S1:** Additional total lifetime cancer risk for males in the respective age groups for all CT scanners used. ‘Optimized protocol’ refers to the last optimization step applied to the corresponding CT scanner.

|  | | | **Initial protocol** | | | **Optimized Protocol** | | |
| --- | --- | --- | --- | --- | --- | --- | --- | --- |
| Age at exposure (years) | Additional Lifetime cancer risk (% per Sv) [[30]](https://www.zotero.org/google-docs/?lgL6yV) | CT scanner | Patients (n) | Mean effective dose (mSv) | Additional lifetime cancer risk  (10^-3^ %) | Patients (n) | Mean effective dose  (mSv) | Additional lifetime cancer risk  (10^-3^ %) |
| 10–19 | 14 | A  B  C  D | 0  2  0  — | —  4.45  —  — | —  62.3  —  — | 0  3  0  0 | —  2.26  —  — | —  31.6  —  — |
| 20–29 | 9.5 | A  B  C  D | 1  3  0  — | 4.46  4.45  —  — | 42.4  42.3  —  — | 0  8  0  0 | —  2.26  —  — | —  21.5  —  — |
| 30–39 | 7.3 | A  B  C  D | 0  6  0  — | —  4.45  —  — | —  32.5  —  — | 4  17  0  1 | 1.44  2.26  —  0.66 | 10.5  16.5  —  4.8 |
| 40–49 | 5.7 | A  B  C  D | 0  12  0  — | —  4.45  —  — | —  25.4  —  — | 6  49  3  0 | 1.44  2.26  1.93  — | 8.2  12.9  11.0  — |
| 50–59 | 4.1 | A  B  C  D | 6  44  3  — | 4.46  4.45  3.66  — | 18.3  18.3  15.0  — | 30  82  2  3 | 1.44  2.25  1.93  0.66 | 5.9  9.2  7.9  2.7 |
| 60–69 | 2.6 | A  B  C  D | 5  79  4  — | 4.45  4.45  3.66  — | 11.6  11.6  9.5  — | 54  177  22  2 | 1.44  2.25  1.93  0.75 | 3.7  5.9  5.0  1.9 |
| 70–79 | 1.4 | A  B  C  D | 12  96  5  — | 4.46  4.45  3.66  — | 6.2  6.2  5.1  — | 45  233  45  5 | 1.44  2.25  1.93  0.69 | 2.0  3.2  2.7  1.0 |
| 80–89 | 0.6 | A  B  C  D | 11  105  6  — | 4.45  4.45  3.66  — | 2.7  2.7  2.2  — | 28  255  25  5 | 1.44  2.25  1.93  0.62 | 0.9  1.4  1.2  0.4 |
| 90–99 | 0.1 | A  B  C  D | 2  17  0  — | 4.46  4.45  —  — | 0.4  0.4  —  — | 6  50  4  0 | 1.44  2.25  1.93  — | 0.1  0.2  0.2  — |
| 100–109 | 0.1 | A  B  C  D | 0  0  0  — | —  —  —  — | —  —  —  — | 0  0  0  0 | —  —  —  — | —  —  —  — |

**Table S2:** Additional total lifetime cancer risk for females in the respective age groups for all CT scanners used. ‘Optimized protocol’ refers to the last optimization step applied to the corresponding CT scanner.

|  | | | **Initial protocol** | | | **Optimized Protocol** | | |
| --- | --- | --- | --- | --- | --- | --- | --- | --- |
| Age at exposure (years) | Additional lifetime cancer risk (% per Sv) [[30]](https://www.zotero.org/google-docs/?eE4BSa) | CT scanner | Patients (n) | Mean effective dose (mSv) | Additional lifetime cancer risk  (10^-3^ %) | Patients (n) | Mean effective dose  (mSv) | Additional lifetime cancer risk  (10^-3^ %) |
| 10–19 | 9.9 | A  B  C  D | 0  1  0  — | —  4.45  —  — | —  44.1  —  — | 0  0  0  0 | —  —  —  — | —  —  —  — |
| 20–29 | 7.5 | A  B  C  D | 0  4  0  — | —  4.46  —  — | —  33.4  —  — | 4  3  0  0 | 1.44  2.25  —  — | 10.8  16.9  —  — |
| 30–39 | 5.5 | A  B  C  D | 0  5  0  — | —  4.45  —  — | —  24.5  —  — | 5  9  0  0 | 1.44  2.25  —  — | 7.9  12.4  —  — |
| 40–49 | 5.1 | A  B  C  D | 0  9  0  — | —  4.45  —  — | —  22.7  —  — | 7  17  0  0 | 1.43  2.26  —  — | 7.3  11.5  —  — |
| 50–59 | 3.3 | A  B  C  D | 2  20  1  — | 4.46  4.45  3.66  — | 14.7  14.7  12.1  — | 48  39  6  1 | 1.44  2.26  1.93  0.53 | 4.8  7.4  6.4  1.7 |
| 60–69 | 1.9 | A  B  C  D | 3  34  5  — | 4.45  4.45  3.66  — | 8.5  8.5  7.0  — | 42  125  15  2 | 1.44  2.26  1.93  0.66 | 2.7  4.3  3.7  1.2 |
| 70–79 | 1.0 | A  B  C  D | 3  88  4  — | 4.46  4.45  3.66  — | 4.5  4.5  3.7  — | 47  192  32  5 | 1.44  2.26  1.93  0.58 | 1.4  2.3  1.9  0.6 |
| 80–89 | 0.3 | A  B  C  D | 9  126  9  — | 4.46  4.45  3.66  — | 1.3  1.3  1.1  — | 51  286  34  11 | 1.44  2.26  1.93  0.55 | 0.4  0.7  0.6  0.2 |
| 90–99 | 0.0 | A  B  C  D | 2  41  4  — | 4.46  4.45  3.66  — | 0.0  0.0  0.0  — | 10  71  5  4 | 1.44  2.26  1.93  0.58 | 0.0  0.0  0.0  0.0 |
| 100–109 | 0.0 | A  B  C  D | 0  1  0  — | —  4.45  —  — | —  0.0  —  — | 0  1  0  1 | —  2.26  —  0.57 | —  0.0  —  0.0 |
